# A Multimodal Imaging Pipeline to Decipher Cell-Specific Metabolic Functions and Tissue Microenvironment Dynamics

**DOI:** 10.1038/s41596-024-01118-4

**Published:** 2025-01-29

**Authors:** Sharavan Vishaan Venkateswaran, Peter Kreuzaler, Catherine Maclachlan, Greg McMahon, Gina Greenidge, Lucy Collinson, Josephine Bunch, Mariia Yuneva

**Affiliations:** 1 The Francis Crick Institute, London, UK; 2 The National Physical Laboratory, Teddington, UK; 3 University of Cologne, Faculty of Medicine and University Hospital Cologne, Cluster of Excellence Cellular Stress Responses in Aging-associated Diseases (CECAD), Cologne, Germany

## Abstract

Tissue microenvironments are extremely complex and heterogenous. It is challenging to study metabolic interaction between the different cell types in a tissue with the techniques that are currently available. In this paper we describe a multimodal imaging pipeline that allows cell type identification and nanoscale tracing of stable isotope labelled compounds. This pipeline extends upon the principles of correlative light, electron, and ion microscopy (CLEIM), by combining confocal microscopy reporter or probe-based fluorescence, electron microscopy (EM), stable isotope labelling and Nanoscale secondary ion mass spectrometry (NanoSIMS). We apply this method to murine models of hepatocellular and mammary gland carcinomas to study uptake of glucose derived carbon (^13^C) and glutamine derived nitrogen (^15^N) by tumour associated immune cells. In vivo labelling with fluorescent-tagged antibodies (B220, CD3, CD8a, CD68) in tandem with confocal microscopy allows for the identification of specific cell types (B cells, T cells and macrophages) in the tumour microenvironment (TME). Subsequent image correlation with electron microscopy offers the contrast and resolution to image membranes and organelles. NanoSIMS tracks the enrichment of stable isotopes within these intracellular compartments. The whole protocol described here would take approximately 6 weeks to perform from start to finish. Our pipeline caters to a broad spectrum of applications, as it can easily be adapted to trace the uptake and utilisation of any stable isotope labelled nutrient, drug, or a probe by defined cellular populations in any tissue *in situ*.

## Introduction

### Development of the protocol

Metabolism is one of the most fundamental aspects of cellular biology, enabling the chemical fluxes to generate energy, whilst providing the building blocks for all biomolecules needed in cell maintenance and propagation. Evaluating the metabolic organisation of complex tissue microenvironments and dissecting interactions between their components is thus vital to decipher cellular function, understand disease mechanisms and design efficient therapeutic approaches. Comprehensive imaging methods for pinpointing metabolic activities in specific cell types at a subcellular level are needed. Our pipeline addresses this gap using a multimodal imaging approach which leverages the strength of pre-existing individual modalities^1^.

Our pipeline builds on established methods: mainly the correlative light, electron microscopy (CLEM) reported by Maclachlan et al.^2^, as well as the combination with secondary ion mass spectrometry (CLEIM) presented by Fearns et al.^3^ and de Boer et al.^4^. Injection of fluorescent antibodies was modelled on mouse studies utilising these methods^5,6^, but adapted for our purposes and made compatible with commercially available antibodies. Labelled metabolites are administered using procedures previously developed and described in our research, e.g. Mendez et al.^7^. Work done by Quinn et al^8^ integrates NanoSIMS with transmission electron microscopy (TEM), while Lechene et al^9^ published a method that utilises multi-isotope imaging mass spectrometry (MIMS) in combination with TEM. However, both these methods do not utilise correlative light microscopy, which is essential for identifying different cell populations within a complex tissue microenvironment. The approach by Loussert-Fonta et al^10^, based on CLEIM, has a lot of similarities to the protocol we describe here but uses TEM instead of scanning electron microscopy (SEM).

Our protocol, however, offers some key advantages and improvements over these previously published methods for a broad range of applications.

SEM provides several benefits over TEM for our purposes. SEM allows imaging of much larger areas, and the elimination of the requirement for electron transparency means more material is available for sputtering. This is particularly important when performing NanoSIMS, because implanting the sample with Cs^+^ from the primary ion beam is required for achieving good ionisation. This process often leaves only a limited amount of sample to sputter for subsequent analysis. When using ^13^C labelling as we do, this can be particularly problematic, especially while looking at small enrichments above natural abundance, as poor counting statistics might impede detection.

Additionally, our method better preserves the ultrastructure compared to using cryofixation and TEM (Loussert-Fonta et al^10^). This is crucial when examining cellular organelles such as mitochondria. Finally, we combine confocal microscopy with volume electron microscopy (vEM) for the entire tissue section, thereby providing a greater target volume for analysis.

There are a few other notable methods employed in the field which are not based on CLEM/CLEIM that have been used to address similar questions, we have listed these in Table 1 along with their respective advantages and limitations.

### Applications of the method

**Figure 1:**
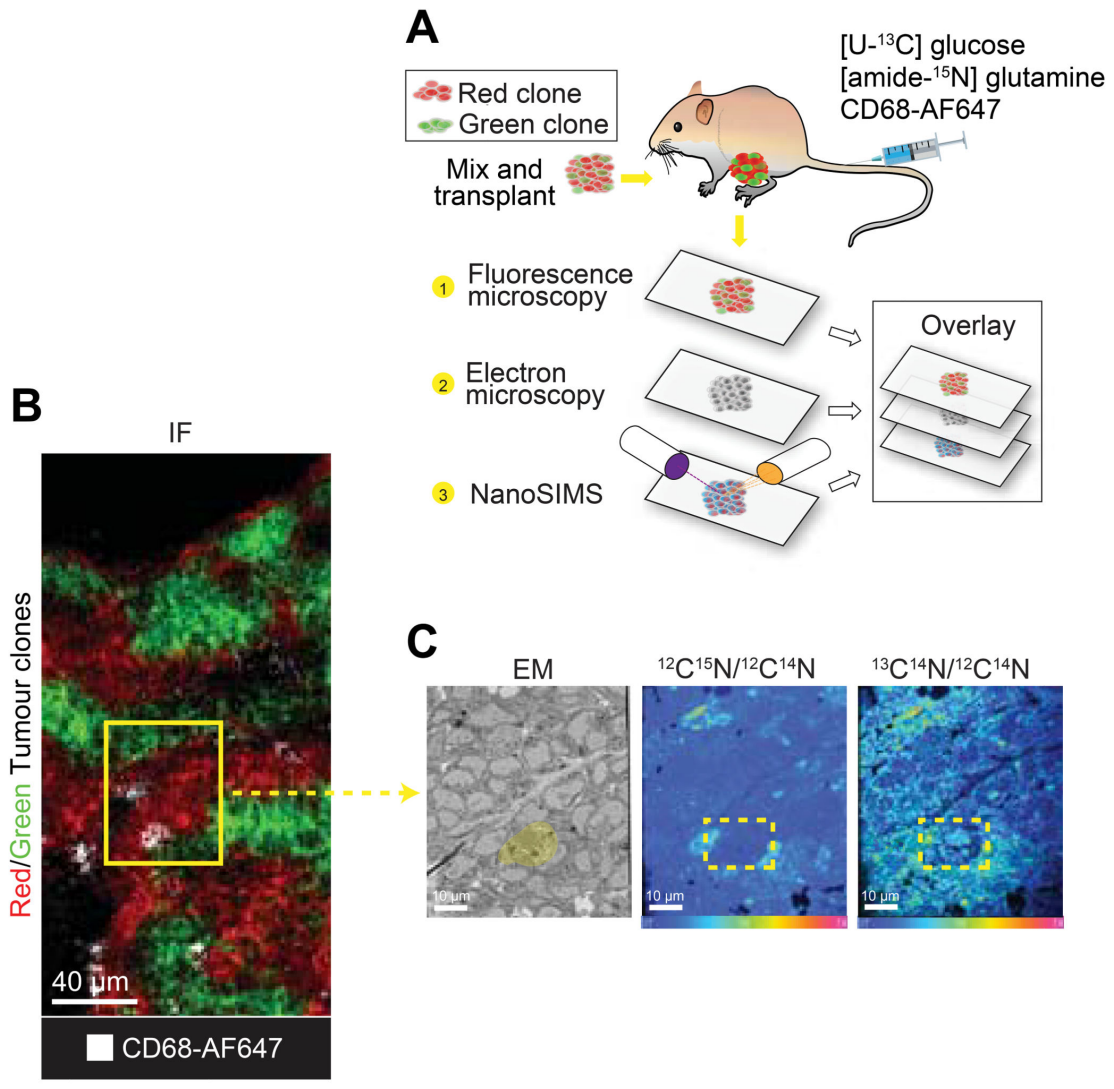
Using correlative fluorescence microscopy, EM and NanoSIMS analysis to evaluate glucose and glutamine catabolism in specific cells of bi-clonal mammary gland tumours. (A) Schematic of inducible and traceable mouse model of heterogeneity in breast cancer and experimental workflow used by Kreuzaler et al^1^. (B) Representative panel of region of interest in the bi-clonal tumour with red clones, green clones and macrophages (CD68) identified with confocal fluorescence image analysis (IF). (C) SEM and correlative NanoSIMS nitrogen/carbon isotope ratio images of region of interest with tumour cells and a macrophage (yellow ROI). Isotope ratio images are displayed as a hue saturation intensity (HSI) transformation. The blue hue represents the natural abundance ratios which are 0.37% and 1.1% respectively, while pink hue represents a value of twice the natural ratio in the ^12^C^15^N/^12^C^14^N and ^13^C^14^N/^12^C^14^N HSI image. Figure adapted from Kreuzaler et al^1^.

In our original publication^1^ we utilised an inducible and traceable model of MYC heterogeneity in breast cancer which we had previously developed and characterised^16^. Briefly, this model enables the creation of triple-negative mammary tumours driven by WNT1, with the optional inclusion of a MYC-ER^T2^ construct. The MYC-ER^T2^ construct expresses supraphysiological levels of the MYC-ER fusion protein, which can be activated by tamoxifen administration. Clones lacking MYC-ER^T2^ express tdTomato (red clone) as a tracer, while clones containing MYC-ER^T2^ express enhanced green fluorescent protein (green clone). Mixing these two clones produces bi-clonal tumours. We initially developed this imaging pipeline to study the metabolic interaction between these two distinct tumour clones in the bi-clonal tumours. Correlative light microscopy was crucial for differentiating the red and green clones within the tumour tissue. The EM provided the necessary contrast and resolution to image membranes and organelles of the tumour cells, while NanoSIMS enabled tracking the enrichment of stable isotopes within these intracellular compartments (Figure 1A)^1^.

We then aimed to validate whether this protocol could be applied to study the metabolic dynamics of other cell types within the TME that lack a fluorescent tracer expression. To achieve this, we combined our imaging pipeline with the in vivo administration of non-depleting fluorescent-tagged antibody (CD68-AF647) to label macrophages in the bi-clonal tumour model (Figure 1B). We were limited to only exploring one additional cell type in this model due to the green and red fluorescent channels being already taken up by the two different tumour clones. The results show the uptake of glucose derived carbon (^13^C) and glutamine derived nitrogen (^15^N) by the macrophage (yellow ROI) and surrounding tumour cells (Figure 1C).

**Figure 2:**
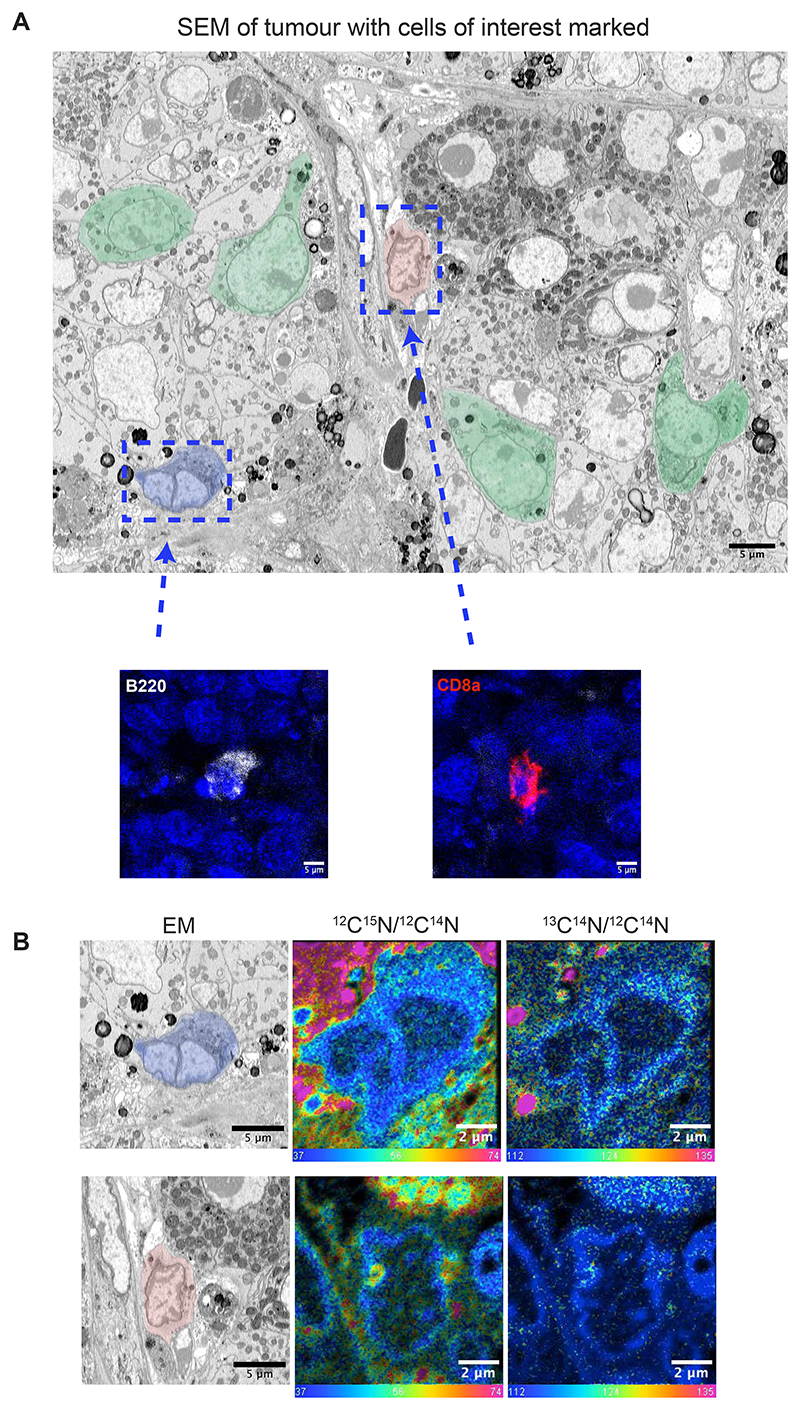
Using correlative fluorescence microscopy, EM and NanoSIMS analysis to evaluate glucose and glutamine catabolism in specific cells of MYC-induced liver tumours. (A) SEM images with cells of interest (B cells – blue ROI, T cells – red ROI, tumour cells – green ROI) – top panel. B and T cells are identified by confocal fluorescence analysis using fluorescently labled anti-B220 and anti-CD8a antibodies, respectively – bottom panel. (B) Correlative NanoSIMS isotope ratio images (^12^C^15^N/^12^C^14^N, middle panel, and ^13^C^14^N/^12^C^14^N, right panel) of the B (blue ROI) and T (red ROI) cells from A. Isotope ratio images are displayed as a hue saturation intensity (HSI) transformation. The blue hue represents the natural abundance ratios which are 0.37% and 1.1% respectively.

To further test the robustness of our protocol, we decided to apply the complete pipeline to a different tumour model, specifically MYC-induced liver tumours^7^. In this model, we examined tumour-resident T and B cells, which were labelled through the in vivo administration of non-depleting fluorescent-tagged antibodies (CD3-AF488 for T cells, CD8a-PE for cytotoxic T cells, B220-AF647 for B cells). Figure 2 shows the uptake of glucose derived carbon (^13^C) and glutamine derived nitrogen (^15^N) by cytotoxic-T cells (CD8a, red ROI) and B cells (B220, blue ROI). These results successfully demonstrate how our protocol can be expanded and applied to study the metabolic dynamics of different cell types within the TME across two distinct tumour models. We propose that the current iteration of this pipeline can be similarly adapted for various applications, providing valuable insights into cellular interactions within cancer and other research areas that utilise preclinical models.

### Limitations

Fluorescent labelling of the cells is done in vivo using fluorescently labelled antibodies. These probes are not able to enter healthy cells, therefore all antibodies must recognise an extracellular epitope.

The number of different cell types that can be detected concurrently is limited by the number of fluorophores that can be imaged simultaneously using a confocal microscope; this is usually 3 biomarkers plus one nuclear counterstain.

The spatial resolution of NanoSIMS typically spans from tens to hundreds of nanometres, defined by the diameter of the primary ion beam used to raster scan and sputter the sample. While this resolution is theoretically adequate for discerning most cell organelles, smaller target organelles or lower metabolite concentrations/ densities will result in a decrease in the quantity of atoms and molecules (measured in yoctomoles) available for ionization within the sub-femtoliter volume under analysis.

NanoSIMS sensitivity can be limited and variable as not all atoms and molecules are ionized during the primary beam induced sputter process. A large fraction of these ejected atoms and molecules retains a neutral charge and thus cannot be effectively collected and steered into the mass spectrometer. Consequently, this limitation often leads to a narrowed dynamic range in detection capabilities^17^.

While NanoSIMS offers organelle level resolution, the technology only allows for measurement of individual atoms (such as C and N) or very simple molecules, this is due to the chemical fixation which causes loss of soluble components within the cells. When infusing with [U-^13^C] glucose for example, we are only able to measure the isotope ratio of ^13^C/^12^C derived from glucose i.e. we do not get any molecular information about the metabolic conversion of the labelled metabolite.

### Experimental design

#### Choice of antibodies

The choice of antibodies depends on the tissue that you are interested in studying and the biological question. Previous research has demonstrated that tumour-infiltrating T cells and B cells play a critical role in determining prognosis and guiding therapeutic intervention in hepatocellular carcinoma (HCC)^18–20^. Based on these studies, we selected antibodies against CD3, CD8a and B220 to, cytotoxic T cells and B cells, respectively, in our MYC-induced liver tumour mouse model.

#### Choice of metabolite // choice of isotope

In addition to the results published in Kreuzaler et al^1^, in this paper, we further demonstrate the strength of our imaging pipeline by presenting another case study—the spatially resolved incorporation of [U-^13^C] glucose-derived carbon (^13^C) and [amide-^15^N] glutamine-derived nitrogen (^15^N) into B cells and T cells within the murine liver TME. We chose these metabolites specifically because we and others in the field have previously shown that glucose and glutamine catabolism is increased in MYC-driven tumours ^7,21^.

NanoSIMS can, however, be used to detect any heavy atom (deuterium, 18O, 13C, 15N, etc.). Based on a biological question, a labelled metabolite catabolised through a defined pathway(s) would give a clearer readout for the pathway activity within specific TME compartments. Indeed, we showed that this strategy is effective not only for glucose and glutamine with tracing the incorporation of nitrogen (^15^N) derived from labelled pantothenic acid (vitamin B_5_) in our previous publication^1^.

#### The in vivo experiment

As described earlier, the bi-clonal mammary gland tumour model enables the generation of triple-negative mammary tumours driven by WNT1, with the optional inclusion of a MYC-ER^T2^ construct. To initially generate spontaneous non-recombined tumours as a source of bi-clonal tumours, Rosa26-CAG-lox-STOP-lox-MYC-ER^T2^/ Rosa26-mTmG/MMTV-Wnt1 mice were used^16^. The MYC-ER^T2^ construct expresses supraphysiological levels of the MYC-ER fusion protein, which can be activated by tamoxifen administration. Clones lacking MYC-ER^T2^ express tdTomato (red clone) as a tracer, while clones containing MYC-ER^T2^ express enhanced green fluorescent protein (green clone). Mixing these two clones produces bi-clonal tumours (Figure 1A)^1^.

MYC-induced liver tumours are generated via hydrodynamic-based transfection to manipulate gene expression in hepatocytes. This is a systemic administration of plasmid DNA in mice, where the plasmids encoding genes of interest are injected through the tail vein^22^.

Once the tumours were generated in both models, the labelled metabolite infusion and antibody injections were performed. Based on our prior experience, we carried out a 3-hour infusion via tail vein for the labelled metabolites to achieve a higher percentage of the label enrichment in a metabolite pool. This also ensures a stronger signal to noise ratio in the NanoSIMS. Tumour resident T and B cells were labelled by non-depleting intravenous (iv) bolus administration of fluorescent-tagged antibodies as specified above.

#### Preparing the microscopy samples and data acquisition

The tumours were then extracted, fixed, and cut into slices using a vibratome. Following which, traditional confocal microscopy was used to detect different cell types in the TME (CD3-AF488 for T cells, CD8a-PE for cytotoxic T cells, B220-AF647 for B cells and CD68-AF647 for macrophages).

**Figure 3:**
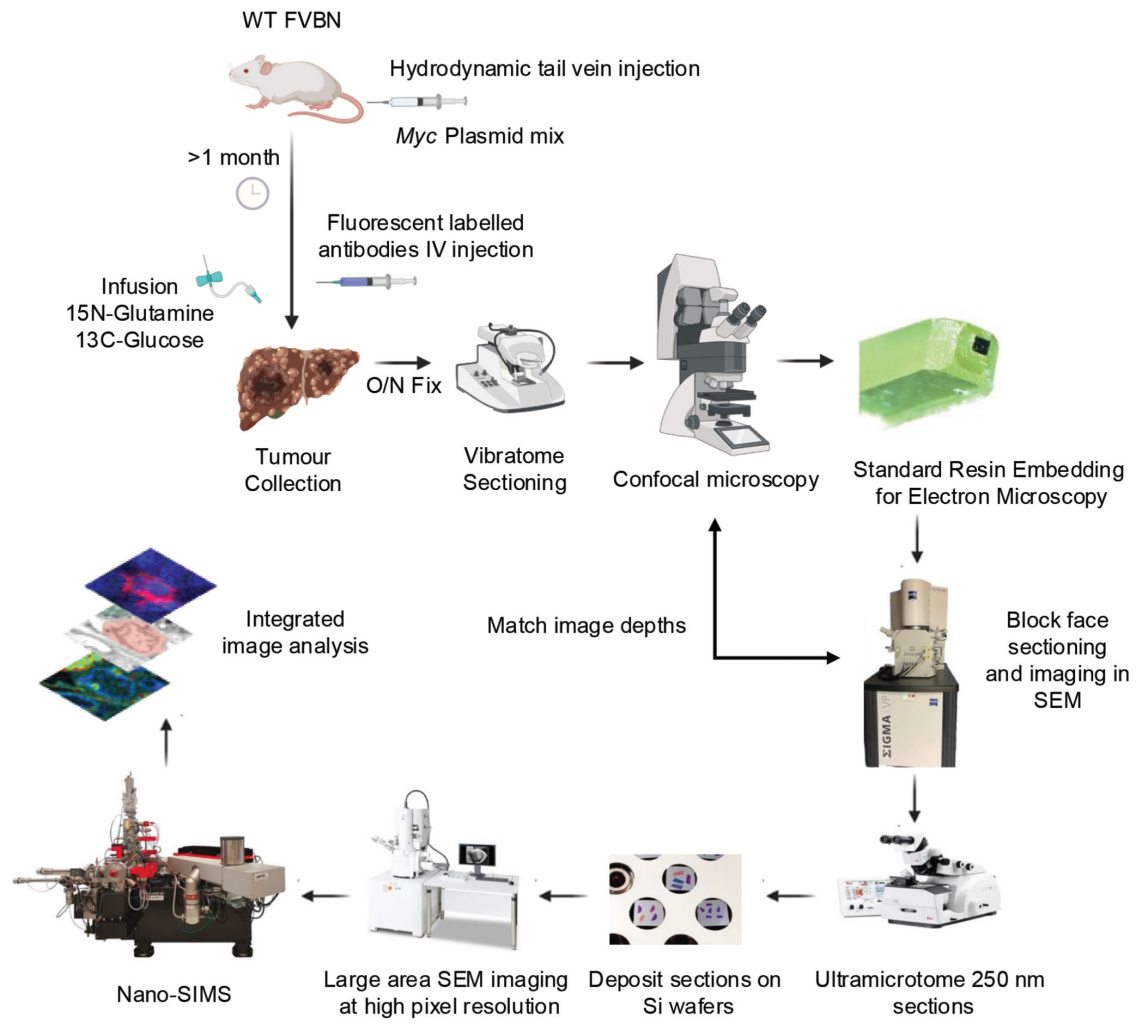
Flow diagram showing the steps of the multimodal imaging pipeline (Created with BioRender.com).

The tissue slices were then prepared for vEM, and the confocal stack was used for targeted trimming to the region of interest (ROI) using serial block face scanning electron microscopy (SBF-SEM). Slices were then cut from the exposed surface of the block using a diamond knife in an ultramicrotome, collected onto substrates, and imaged in array tomography format in the SEM, prior to serial spatial metabolic imaging using NanoSIMS, helps us to correlate the metabolite derived label to subcellular structures (Figure 3).

NanoSIMS enabled us to measure the ^13^C^14^N/^12^C^14^N and/or ^13^C/^12^C isotope ratio, derived from [U-^13^C] glucose, and the ^12^C^15^N/^12^C^14^N and/or ^15^N/^14^N isotope ratio, derived from [amide-^15^N] glutamine, offering an insight into the metabolic activities in different cells and cellular compartments within the TME^23^.

#### ^23^Quantitation and standards

We would recommend a minimum of 2 biological replicates (mice/tumours) per experimental group for this protocol. The specific number of biological replicates will be dependent on the biological question. In our original paper 2 biological replicates was sufficient to validate our hypothesis because with single cell resolution and relatively large areas covered by correlative light microscopy and SEM, we can analyse multiple cells of interest within the same tissue^1^. We prepared consecutive sections on wafer for immediate technical replicates. However, due to the precise nature of the method we did not have any loss of signal and were able to measure the isotope ratios in multiple regions within individual cells, hence it was not necessary to rely on the technical replicates.

This method is however not yet capable of absolute quantification of the amount of ^13^C or ^15^N and is only a ratio metric comparison. As we are not reporting any type of absolute quantification, the protocol does not require any analytical standards, but should the protocol be adapted to determine the level of a drug, for example, denoted by an isotopic signature, then a matrix matched standard would be required to convert secondary ion count rate into an absolute concentration value.

#### Method modification and optimisation

Adopting this protocol for other biological applications should be straightforward. Optimisation would be required for the accurate detection of the specific labelled metabolites/compounds chosen and the respective cell types of interest using the relevant non-depleting fluorescent-tagged antibodies. This could be done using tissue collected from wild-type mice without tumours to test different labelled compounds and antibody incorporation in the respective tissue of interest. Positive and negative controls can be picked depending on the experiment. In our case for a negative control, we collected a tumour tissue from the MYC-induced liver tumour model without the administration of [U-^13^C] glucose and [amide-^15^N]. The negative control was used to establish the ratio prior to analysing the tumours with the labelled glucose and glutamine. In most cases, due to the nature of the method being a comparison of ratios, the positive and negative controls may not be strictly necessary.

In the following sections, we describe the methodologies employed in each imaging technique and a step-by-step guide to our multimodal approach.

### Expertise needed to implement the protocol

A certain degree of expertise and training is required for work involving animals. The specific licence required depends on the country in which the experiments are performed, and the techniques involved. For example, hydrodynamic tail vein injection described in this case study is a difficult technique to perform and requires considerable effort to become proficient. But this pipeline does not strictly require this, and the methods described here can be easily applied to a wide array of mouse models as well as other in vivo studies. Several genetically engineered mouse models such as spontaneous tumour models, for example, do not require any such technical proficiency to generate tumours. Large institutes, universities, and companies have core facilities responsible for the handling and care of experimental animals. Such facilities can help researchers design and set up experiments involving animals.

EM is a common method, with robust protocols for embedding and processing. Consequently, 2D EM followed by NanoSIMS, which is performed on the very same surface as EM and consequently requires identical preparation, can readily be performed by any researcher or facility with experience in sample preparation for EM. Conversely, the correlation with volumetric fluorescent imaging for ROI selection will require targeting of the ROI with an SBF-SEM, followed by array tomography prior to NanoSIMS acquisition. Both techniques will require collaboration with a well-equipped core facility experienced in volume EM and correlative workflows.

Setting up NanoSIMS at any laboratory poses several challenges due to its reliance on expensive instruments and high level of technical proficiency for its operation and maintenance. Consequently, NanoSIMS is typically performed in core facilities or shared instrumentation settings equipped with the requisite expertise and resources. These facilities offer access to cutting-edge instrumentation, expert technical assistance, and comprehensive training to aid researchers in experiment design and data analysis. Collaborating with such core facilities presents a cost-effective and efficient approach, especially for researchers with intermittent requirements for NanoSIMS technology^17^.

## Materials

### Biological materials

- The method for generation of bi-clonal mammary gland tumours has been described in detail previously^1,16^. The liver tumour model is described in the Procedure of this protocol.
- **Mice**. Adult (7- to 9-week-old) male mice of FVB/N strain (RRID:MGI:2160001) from The Francis Crick Institute Biological Research Facility (BRF).

<CRITICAL> All procedures and animal husbandry were carried out in accordance with the UK Home Office, under the Animals (Scientific Procedures) Act 1986, and the Crick Animal Welfare and Ethical Review Body (AWERB), which is delivered as part of the BRF Strategic Oversight Committee (BRF-SOC), under the Project Licence number P609116C5.

<CAUTION> The use of experimental mice must be in compliance with national and institutional regulations related to the use of animals for research purposes. Permissions to carry out experiments should be obtained before the start of animal studies.

### Reagents

- Sterile saline solution, 0.9% NaCl (40120975, bioWORLD)
- Isoflurane -Vet 100% w/w Inhalation Vapour, Liquid (Merial, Boehringer Ingelheim)
- DPBS 1x (Gibco)
- Agarose, Low gelling temperature (1002718026, Sigma-Aldrich)
- DAPI (4’,6-Diamidino-2-Phenylindole, Dihydrochloride) (D1306, Invitrogen)
- VALAP - Mixture of Vaseline, Lanolin and Paraffin (1:1:1 w/w/w)
- Leica Microsystems Immersion Oil for Microscopes (12847995, Fisher Scientific)

#### Isotope labelled nutrients

- [U-^13^C] glucose (CLM-1396-PK, Cambridge Isotope Laboratories)
- [amide-^15^N] glutamine (NLM-557-PK Cambridge Isotope Laboratories).

#### Plasmids –

- pT3-EF1α-c-MYC (RRID:Addgene_92046, hhttps://www.addgene.org/92046/)
- pT3-EF1α-MCL1 (RRID:Addgene_133299; https://www.addgene.org/133299/, RRID:Addgene_117726; https://www.addgene.org/117726/)
- pCMV-SB (RRID:Addgene_24551, https://www.addgene.org/24551/).

#### Antibodies –

- CD8a-PE (Thermo Fisher Scientific Cat# 12-0081-82, RRID:AB_465530, https://scicrunch.org/resolver/RRID:AB_465530)
- CD3-AF488 (Thermo Fisher Scientific Cat# 53-0032-82, RRID:AB_2848414, https://scicrunch.org/resolver/RRID:AB_2848414)
- B220-AF647 (BioLegend Cat# 103202, RRID:AB_312987, https://scicrunch.org/resolver/RRID:AB_312987)
- CD68-AF647 (BioLegend Cat# 137003, RRID:AB_2044001, https://scicrunch.org/resolver/RRID:AB_2044001)

#### Reagents for EM

**<CRITICAL>** The chemicals required for preparing these solutions are listed in the next section.

- Solution X (Na_2_HPO_4_•2H_2_O; dihydrate solution) stored at 4ºC
- Solution Y (NaH_2_PO_4_•H_2_O; monohydrate solution) stored at 4ºC
- 0.1 M phosphate buffer (PB) at pH 7.4
- 0.2M phosphate buffer (PB) at pH 7.4
- 2% reduced osmium – made fresh
- 1% uranyl acetate – stored at 4ºC
- Lead aspartate (pH 5.5) - made fresh
- 1% thiocarbohydrazide – made fresh
- 4% EM-grade formaldehyde in 0.1M PB – made fresh
- 4% EM-grade formaldehyde, 2.5% glutaraldehyde in 0.1M PB – made fresh
- 3% potassium ferricyanide – stored at 4ºC
- Ethanol dehydration series; 30%, 50%, 70%, 90%, 100% (dry) ethanol
- Durcupan resin – made fresh
- 0.03M aspartic acid pH 3.8 – stored at 4ºC
- 1M potassium hydroxide (KOH)

#### Chemicals for EM

- 36% EM-grade formaldehyde (F003, TAAB laboratories)
- 25% glutaraldehyde (G004, TAAB laboratories)
- di sodium hydrogen phosphate dihydrate (Na_2_HPO_4_•2H_2_O; dihydrate) (28029.260, VWR Chemicals)
- Sodium phosphate monobasic monohydrate (NaH_2_PO_4_•H_2_O; monohydrate) (102633600, Sigma-Aldrich)
- 4% osmium tetroxide (O011, TAAB laboratories)
- Thiocarbohydrazide (TCH) (102053096, Sigma-Aldrich)
- Uranyl acetate (R1260A, Agar Scientific)
- Potassium ferricyanide (P018, TAAB laboratories)
- Distilled water
- L-aspartic acid (A4534-100G, Sigma-Aldrich)
- Lead (II) nitrate (102475122, Sigma-Aldrich)
- Potassium hydroxide (P1767-250G, Sigma-Aldrich)
- Ethanol (10680993, Fisher Scientific)

#### Durcupan resin components

- ACM single component A (Sigma Aldrich 44611)
- ACM single component B (Sigma Aldrich 44612)
- ACM single component C (Sigma Aldrich 44613)
- ACM single component D (Sigma Aldrich 44614)

### EQUIPMENT

#### Materials for EM

- Filter units, 500ml (for PB and aspartic acid) (151-4020, Thermo Scientific)
- Metal pin (10-006002-50, Labtech)
- Sable hairbrushes (AGG3446, Agar Scientific)
- Silver epoxy (604057, CW2400 adhesive, Farnell)
- Platinum disc target, 57mm dia x 0.1 mm, Coater type 1 (AGB7392, Agar Scientific)
- Silicon wafers (G3390, Agar Scientific)
- SEM stubs (10-002012-100, Labtech Electron Microscopy)
- Eyelash tool (Single eyelash attached to a cocktail stick using nail varnish)
- Adhesive carbon tabs (15-000412, Labtech Electron Microscopy)
- ACLAR sheets (AGL4458, Agar Scientific)

#### Consumables and kits

- Endotoxin-free Maxiprep kit (12362, Qiagen)
- Pierce concentrator 10K MWCO (88513, Thermo Fisher Scientific)
- Insulin syringe 0.5ml, 29G, 12.7mm (BD)
- Tail vein catheter (504147, World Precision Instruments)
- SuperFrost Plus Adhesion slides (12312148, Fisher Scientific)

#### Equipment and imaging systems

- Centrifuge (Fresco 21, Thermo Fisher Scientific)
- Vaportec isoflurane vaporiser (Burtons Veterinary, UK)
- Aladdin AL-1000 pump (World Precision Instruments)
- Vibrating knife ultramicrotome (VT1200S, Leica)
- Confocal microscope (Leica, Falcon SP8)
- Stereo microscope (MC205C stereo, DMC 4500 Camera, Leica Microsystems)
- Ultramicrotome (EM UC7, Leica Microsystems)
- Rotary-pumped sputter coater (Q150S, Quorum Technologies, Lewes, UK)
- Serial block face scanning electron microscope (SBF-SEM) - consisting of a 3View2XP (Gatan, Pleasanton, CA) attached to a Sigma VP SEM (Zeiss, Oberkochen, Germany) with focal charge compensation (FCC, Zeiss, Oberkochen, Germany)
- Histological diamond knife (DiATOME, Nidau, Switzerland)
- Quanta 250 FEG SEM (Thermo Fisher Scientific, Waltham MA USA)
- Low voltage high-contrast backscattered electron detector (vCD, Thermo Fisher Scientific, Waltham MA USA)
- NanoSIMS (Cameca NanoSIMS 50L, Cameca/Ametek, Gennevilliers, France)

#### Software

- Photoshop (Adobe Inc., San Jose, CA USA)
- Leica Application Suite X (LAS X) (Leica Microsystems)
- TrackEM2 plugin for Fiji (https://fiji.sc/, https://imagej.net/plugins/trakem2/)
- Digital Micrograph (version 2.3.2.888, Gatan)
- SBEMImage (https://github.com/SBEMimage)^24^.
- xT Microscope control (version 6.2.8, ThermoFisher Scientific)
- MAPS software (ThermoFisher Scientific)
- OpenMIMS plugin for Fiji (https://fiji.sc/, https://github.com/BWHCNI/OpenMIMS)
- BigWarp plugin for Fiji (https://imagej.net/plugins/bigwarp)^25^.

### Reagent setup

#### VALAP

1. Measure out a blend of petroleum jelly (Vaseline), lanolin, and paraffin in equal parts (1:1:1 w/w/w).
2. Heat the mixture in a glass or ceramic container on a hot plate using medium to low heat until completely liquefied.
3. Apply the molten wax mixture onto a glass slide; it should smoothly spread and promptly dry.
4. If rapid solidification occurs, incorporate additional petroleum jelly and lanolin.
5. If slower solidification is observed, add more paraffin.
6. VALAP solidifies at room temperature; prior to use, gently warm it on a hot plate at a low setting^26^.

#### Solution X

Dissolve 18.69 g Na_2_HPO_4_•2H_2_O; dihydrate (VWR 28029.260) in 525 ml ddH_2_O. Filter, sterilise and store at 4º C. It is stable under these conditions for up to a year.

#### Solution Y

Dissolve 13.80 g NaH_2_PO_4_•H_2_O; monohydrate in 500 ml ddH_2_O. Filter, sterilise and store at 4ºC. It is stable under these conditions for up to a year.

#### 0.2M PB

Prepare 0.2M PB by mixing:

Solution X 202.5 ml

Solution Y 47.5 ml

Store at 4ºC for up to a year

#### 0.1M PB

Prepare 0.1M PB by mixing:

Solution X 202.5 ml

Solution Y 47.5 ml ddH_2_O 250 ml

Store at 4ºC for up to a year

#### [H3] 4% formaldehyde in 0.1M PB (Primary fixation)

36% formaldehyde 1.1 ml

0.2M PB 5 ml

ddH_2_O 3.9 ml

Make fresh

#### 4% formaldehyde, 2.5% glutaraldehyde in 0.1M PB (Secondary fixation)

36% formaldehyde 1.1 ml

25% glutaraldehyde 1 ml

0.2M PB 5 ml

ddH_2_O 2.9 ml

Make fresh

#### 2% reduced osmium (2% OsO_4_, 1.5% K_3_Fe(CN)_6_). Safety is critical

In a fume hood, mix equal volumes 4% Osmium Tetroxide and 3% Potassium Ferricyanide

4% Osmium 5 ml

3% Potassium Ferricyanide 5 ml

Make fresh

#### 1% TCH solution

Make fresh ˜10 ml of 1% TCH solution. You will need approximately:

ddH_2_O 10 ml

Thiocarbohydrazide 0.1 g

1. Zero balance with an empty tube.
2. Transfer tube to a fume hood, then add some thiocarbohydrazide powder to the tube.
3. Close the tube, remove from fume hood, and weigh it on the balance.
4. If more powder is needed, repeat steps 2-3 (i.e., only opening the tube and handling powder in the fume hood).
  **<CRITICAL>** For convenience, rather than weighing out exactly 0.1 g, weigh what you added to the tube and adjust the water accordingly. For example, for 0.11 g of thiocarbohydrazide, use 11 ml of water.
5. In the fume hood, add water, close tube.
6. Incubate for 1 h, 60ºC.
7. Swirl to mix every 10 minutes to facilitate dissolving.
8. Filter through a 0.22 µm Millipore syringe filter right before use.

#### 1% aqueous uranyl acetate. Safety is critical

Make ˜50 ml of 1% UA in double distilled water. You will need:

ddH_2_O 50 ml

uranyl acetate 0.5 g

1. Zero a balance with an empty 50 ml tube.
2. Transfer tube to a fume hood, then add some uranyl acetate powder to the tube.
3. Close the tube, remove from fume hood, and weigh it on the balance.
4. If more powder is needed, repeat steps 2-3 (i.e., only opening the tube and handling powder in the fume hood).
5. In the fume hood, add the appropriate amount of water, close the tube and seal with Parafilm.
6. Mix on a vortex for 1-2 min
7. Wrap the tube in aluminium foil to protect from light and place the tube on a rolling mixer for 2-3 h, repeating vortex as above every hour or so, until the uranyl acetate is dissolved.
8. Store at 4ºC. It should be stable for at least 100 days as long as the uranyl acetate stays in solution.

**<CRITICAL>** For convenience, rather than weighing out exactly 0.5 g, weigh what you added to the tube and adjust the water accordingly. For example, for 0.46 g of uranyl acetate, use 46 ml of water.

#### 1M Potassium hydroxide (KOH)

Make 100ml of 1M KOH. You will need:

Potassium hydroxide 5.61g

ddH_2_O 100ml

Store at room temperature, it should be stable for a year as long as the Potassium Hydroxide stays in solution.

#### 0.03M Aspartic acid pH 3.8

Make 250ml of 0.03M aspartic acid pH 3.8. You will need:

L-aspartic acid 1g ddH_2_O 250ml

1. Add aspartic acid powder to water and stir with a magnetic stirrer bar; a small amount will dissolve, which lowers the pH to around 3.
2. pH to approximately 3.8 by adding 1 M KOH dropwise.
3. This lets more aspartic acid dissolve, which lowers the pH again.
4. Wait until the pH stops reducing.
5. Then repeat steps 2-4 until certain all the aspartic acid has dissolved.
6. pH should now remain stable at 3.8.
7. Filter, sterilise and store at 4ºC for up to 2 months.

#### Lead Aspartate

Make 0.66% lead nitrate in 0.03 M pH 3.8 aspartic acid. You will need:

0.03 M pH 3.8 aspartic acid 20 ml

lead nitrate 0.132 g

1. Zero balance with an empty tube.
2. Transfer tube to a fume hood, then add some lead nitrate powder to the tube.
3. Close the tube, remove from fume hood, and weigh it on the balance.
4. If more powder is needed, repeat steps 2-3 (i.e., only opening the tube and handling powder in the fume hood).
5. Transfer to a fume hood and dissolve the lead nitrate in room temperature 0.03 M pH 3.8 aspartic acid stock.
6. Using a glass pipette, pH to 5.5 dropwise with 1 M KOH, swirling gently to mix after each drop, while checking pH. Check carefully that the solution remains clear, and no precipitate has formed.
7. Incubate in glass vial for 30 min 60°C.
  **<CRITICAL>** For convenience, rather than weighing out exactly 0.132 g, weigh what you added to the tube and adjust the water accordingly. For example, for 0.130 g of lead nitrate, use 19.70 ml of water (i.e., to make 0.66% w/v).
8. Make fresh.

**<CRITICAL>** Precipitation can occur easily, more so as pH approaches 5.5; this gives a slightly white, turbid, appearance, rather than the correct, clear liquid. Adding small amounts (1-2 drops) of 1 M KOH will cause slight cloudiness, but this can be dissolved by swirling to mix. Adding more 1 M KOH at once can cause precipitation that cannot be re-dissolved. Do not use magnetic stirrer, the slightest bumps can cause precipitation, even at lower pH.

**<CRITICAL>** During incubation at 60°C no precipitation should form. If you notice any precipitation, discard the solution and start again.

### Durcupan resin

1. Place a stirrer in a plastic beaker.
2. Weigh out the amounts below, using 3 ml plastic Pasteur pipettes with the ends cut off (zero after each weighing), in the fume hood. *The range for Component D comes from a protocol of the National Centre of Microscopy and Imaging Research^27^; larger volumes have been recalculated to give the same range.

**Table:** 

Component A (g)	Component B (g)	Component C (g)	Component D (g)
11.4	10	0.3	0.05-0.1*
22.8	20	0.6	0.125-0.175
34.2	30	0.9	0.2-0.25
3. After adding component D, stir gently by hand to mix the components, then place on a stirrer plate, cover, and stir for at least 10 min at low speed to avoid air bubbles.
4. Stop stirring and leave for 5-10 min for air bubbles to dissipate.
5. Make fresh.

### Equipment setup

#### Confocal Microscope (Leica, Falcon SP8)

- Switch on the microscope and computer as per standard instructions. Start up the LAS X software with the following configuration: machine with 440 pulsed, microscope – DMi8, Stage calibration enabled.
- Set the laser configuration as follows – WLL and 405 lasers set at 70%.
- Under the Acquisition panel, set the excitation and emission ranges for the two PMT detectors, HyD1 and HyD3 for imaging DAPI (358-461nm), Alexa Fluor 488 (448-496nm), PE (565-574nm) and Alexa Fluor 647 (650-665nm) respectively.
- Use the LAS X navigator to locate and mark the sample boundaries under a 20X air objective (HC PL APO 20x/0.75 IMM CORR CS2).
- Utilize the spiral function can be to perform a low-resolution scan to find regions of interest.
- Navigate to the focus map feature to mark the different focal points and corresponding Z stack prior to scanning.
- Use mosaic merge to stitch the tile scan together to obtain a single overview image of the sample.
- When switching to the 63X objective (HC PL APO 63x/1.40 OIL CS2) a drop of immersion oil is applied on the coverslip.

#### Serial Block Face Scanning Electron Microscope

(consisting of a 3View2XP (Gatan, Pleasanton, CA) attached to a Sigma VP SEM (Zeiss, Oberkochen, Germany) with focal charge compensation (FCC, Zeiss, Oberkochen, Germany)

1. Start Digital micrograph (Gatan) and SmartSEM (Zeiss) softwares.
2. Load the sample into the microscope as per instructions.
3. Adjust the focal charge compensator (FCC) to optimal position for the sample.
4. Pump microscope to vacuum.
5. Turn on the SmartSEM (Zeiss) at 2 kV, with an aperture of 30 µm and an FCC level of 50%. Use this as a start point and adjust to suit the sample.
6. In Digital Micrograph, launch the open-source software SBEMimage.
7. Use the Gatan 3view BSD detector to collect back scattered electrons.

#### Scanning Electron Microscope (Thermofisher, Quanta 250 FEG SEM)

1. Launch xT microscope control (v6.2.8).
2. Load the sample as per manufacturer’s instructions.
3. Pump microscope to vacuum.
4. Turn on the beam at 2.5kV and a spot size of 3. Use this as a start point and adjust to suit the sample.
5. Once sample is in focus, launch MAPS software.
6. Image the sample using the vCD (Thermofisher) to collect back scattered electrons.

#### NanoSIMS (Cameca NanoSIMS 50L)

1. Mount sample (sections on 5mm x 5mm Si chips) onto Harvard holder.
2. Load the sample into to the instrument per manufacturer’s instructions and analysis
3. Pump the chamber to 2-5x10-^10^ mbar.
4. Set the z-height of the sample stage.
5. Implant Cs^+^ ions in the region of interest.
6. Position the detectors to the correct radii for analysis.
7. Measure the pulse height distributions of the electron multiplier (EM) detectors and adjust their voltage gains and thresholds if necessary.
8. Examine the detectors further by measuring the C^-^ and CN^-^ count rate on adjacent detectors and use it to measure the ^13^C/^12^C and ^12^C^15^N/^12^C^14^N isotope ratios. This step is imperative for measurement of accurate isotope ratios.
9. Align the secondary column.
10. Optimise the Mass Resolving Power (MRP) to ensure high mass resolution.
11. Acquire high mass resolution spectra for each mass and carefully select the secondary ion position from those scanned through the exit slit. Select the image acquisition parameters depending upon the required spatial resolution, sensitivity, and mass resolution. Further details can be found in McMahon & Lechene^28^.

## Procedure

**<CRITICAL>** In this section we will go through the different steps of the protocol shown in the workflow in detail (Fig 3). The section is further subdivided as follows: hydrodynamics-based transfection of DNA in the liver to generate liver tumours (Steps 1-3), antibody clean-up (Steps 4-7), stable isotope labelling and in vivo administration of antibodies (Steps 8-12), embedding for EM and immunofluorescence imaging (Steps 13-28), targeted single section large area montaging (Steps 29-41), NanoSIMS acquisition (Steps 42-48) and NanoSIMS analysis (Steps 49-51).

### Hydrodynamics-based transfection of DNA in the liver to generate liver tumours *Timing 4 weeks*

**<CRITICAL>** Liver tumours were generated as described previously^7^. Hydrodynamic-based transfection was performed as established^22^, with some variations detailed below.

1. Prepare a mixture comprising 5 μg of pT3-EF1α-c-MYC, 5 μg of pT3-EF1α-Mcl1, and 0.2 μg of sleeping beauty transposase (SB) plasmid DNA (purified using an Endotoxin-free Maxiprep kit) in a 25:1 ratio diluted in a volume of saline equivalent to 10% of the mice’s body weight.
  **<CRITICAL STEP>** To counteract MYC-induced apoptosis and enhance the efficiency of MYC-induced tumorigenesis, the ectopic expression of MYC through hydrodynamics-based transfection was combined with MCL1 expression. By carefully titrating the amount of SB used, we ensured a consistent level of integration events and induction of tumorigenesis.
2. Put the mice under isoflurane anaesthesia using a Vaportec Isoflurane Vaporiser and inject the mixture into the lateral tail vein of the mice (7- to 9-week-old male FVB/N) within 8 to 12 seconds. Place the mice in a ventilated recovery unit overnight following the procedure.
3. Regularly check for the presence of liver tumours by gentle palpitation of the abdominal region of the mice, starting from 2 weeks post injection.
  - You can identify the liver tumours as hard masses in the soft abdominal area or by a swollen abdomen when the tumour progresses further.
  - When there is a 20% increase in the normal abdomen diameter it is considered a humane endpoint. Tumours take an average of 3-4 weeks to reach this stage.
  - Tumours can also be monitored using non-invasive in vivo imaging methods such as ultrasound imaging or MRI (Magnetic Resonance Imaging).

### Antibody clean-up *Timing 20 minutes*

4 Pipette the appropriate volume for 4 μg of each antibody into the concentrator sample chamber of Pierce Concentrator 10K MWCO spin tube.
5 Add 500ul of dPBS into the chamber of the spin tube.
6 Centrifuge the tube at 15000 x g for 10 minutes at 4°C.
7 Discard the flow through and adjust the volume of the purified antibody in the chamber to 40ul with saline for the injection.

### Stable isotope labelling and in vivo administration of antibodies *Timing 4-5 hours*

**<CRITICAL>** The injection of antibodies (step 9) and the initial bolus of the isotope-labelled metabolites (step 11) can be administered as a single injection by mixing both solutions. The total volume for the injection should not exceed the volume allowed in an animal licence.

The advantage of doing this is mainly to reduce the time taken to prepare and administer multiple injections.

8 Once liver tumours are detected as described in step 3, weigh the mice and put them under isoflurane anaesthesia to prepare for injection of antibody suspension and isotope-labelled metabolites.
9 Cannulate the tail vein and affix the tail catheter in place with an adhesive. Use a 0.5ml insulin syringe to inject the purified antibody suspension from step 7 via tail vein injection by attaching the syringe to the catheter tube.
10 Prepare a saline solution consisting of both [U-^13^C] glucose (96 mg/ml) and [amide-^15^N] glutamine (40 mg/ml).
11 Take an appropriate volume of the solution from the previous step for a final concentration of [U-^13^C] glucose at 0.442 mg per g of body weight of the mouse and [amide-^15^N] glutamine at 0.187 mg per g of body weight. Administer the mice with this initial bolus of the solution with both [U-^13^C] glucose and [amide-^15^N] glutamine via tail vein injection using a 0.5ml insulin syringe as described in step 9.
12 Fill a 1 ml insulin syringe with an appropriate volume of the solution from step 10 and set up a 3 h infusion of glucose (0.012 mg per g body weight per minute) and glutamine (0.005 mg per g body weight per minute), maintained at a rate of 0.2 ml h^–1^ on the pump.
  **<CRITICAL STEP>** Infusions of stable isotope mixture is performed through a tail vein catheter utilizing the Aladdin AL-1000 pump. The syringe with the labelled metabolites is set up on the pump and the free end of the teil vein catheter tubing is attached to the needle of the syringe. The flow rate and duration is adjusted on the pump. The infusion protocol was established based on prior experiments^1^.
13 After the end of the infusion, cull the mice by cervical dislocation or other approved S1K methods.
14 Dissect out tumours using sterile surgical tools. Cut large tumour tissues into smaller chunks of approximately 1cm in diameter.

### Fluorescence imaging and sample preparation for EM *Timing 4 days*

15 Fix the tumours overnight in 2-5ml (enough to cover the tumour chunks) of freshly prepared 4% paraformaldehyde in 0.1 M PB at pH 7.4 and store them at 4°C.

**<PAUSE POINT>** After overnight fixation the tumours can be transferred to 0.1M PB and stored at 4°C for extended periods.

16 Following initial fixation, embed the samples in 2% low melting point agarose in 0.1 M PB. Add enough to cover the entire tumour chunk.
17 Use a vibrating knife ultramicrotome with speed of 1 mm/s and an amplitude of 0.75 mm, to collect 150 μm thick sections. Remove excess agarose from the sections and store the tissues in a 24-well plate in 0.1 M PB at 4°C.

**<CRITICAL STEP>** A detailed training guide on how to perform the sectioning has been prepared by our team – https://vimeo.com/763353109

18 Stain the tumour sections with 200 μl DAPI (1:1000 dilution in 0.1M PB – 1µg/mL working concentration) for 30 min. This step can be done in a 24-well plate.
19 Transfer the sections onto a glass slide carefully using a paintbrush, coverslip in 0.1M PB and seal the edges with VALAP.

**<CRITICAL STEP>** Be careful not to damage the section when transferring and to avoid air bubbles which could dry out the section. The section must be in 0.1M PB during the whole imaging session. Take a picture of the orientation of the section on the slide which can help with orientation of the image under the microscope.

20 Image the whole section in a single plane with a Leica SP8 Falcon confocal microscope at a 20X magnification, the tissue boundaries and focal plane can be determined using the LAS X navigator as described in the equipment setup.
21 With the help of the 20X overview scan, choose appropriate regions of interest (ROI) within the tumour section and image a Z-stack of approximately 70 μm depth with a step-size of 2.75 μm at 63X magnification over an area of approx. 1.5 mm^2^. Note the location coordinates for each of the ROIs (regions of interest) within the tumour precisely.
22 After imaging the sections, gently scrape the VALAP from the edges and float the coverslip off by immersing the slide in 0.1M PB.
23 Carefully remove the imaged sections using a paintbrush and transfer them back into the 24-well plate with fresh 0.1M PB. The sections can then be embedded using a protocol adapted from the NCMIR method^27^.
24 Post-fix sections in 4% paraformaldehyde/ 2.5% glutaraldehyde in 0.1 M PB pH 7.4 for 1 h at room temperature.
25 Wash sections in 0.1 M PB (5 x 3 min) before post-fixing in 2% reduced osmium (2% osmium tetroxide / 1.5% potassium ferricyanide) at 4°C for 1 h.
26 Wash the sections in dH_2_O (5 x 3 min), stain in 1% thiocarbohydrazide for 20 min at room temperature.
27 Wash the sections again in dH_2_O (5 x 3 min) and stain in 2% osmium tetroxide for 30 mins at room temperature.
28 Wash the samples once more in dH_2_O (5 x 3 min) and incubate overnight in 1% uranyl acetate at 4°C.
29 The following day wash the sections in dH_2_O (5 x 3 min) and stain en bloc with lead aspartate (pH 5.5) for 30 min at 60°C
  <CRITICAL STEP> It is essential that this step is performed as described in order to get enough contrast in the SBF-SEM).
30 After a final wash in dH_2_O (5 x 3 min), dehydrate sections using a graded series of ethanol (20%, 50%, 75%, 90%, 100% x 2, 20 min each).
31 Perform infiltration with Durcupan resin and ethanol (1:1 resin: ethanol) overnight and then with 100% Durcupan resin for 24 h.
32 Flat embed the sections between two sheets of aclar and place in an oven to polymerise at 60°C for 48 h.

<**CRITICAL STEP>** If Samples are not flat embedded, you will not be able to follow the microscope sample preparation steps.

### Targeted single section large area montaging *Timing* 2-3 days (depending on depth of area of interest)

33 Remove the polymerised sections from the Aclar and prepare blocks from the ROI.

**<CRITICAL STEP>** The ROI is identified by aligning the overview of 63X confocal image of the whole section to an overview image of the now embedded section, acquired using a stereo microscope in Photoshop. This ROI includes the cells of interest marked on the confocal image using the respective markers: CD3-AF488 for T cells, CD8a-PE for cytotoxic T cells and B220-AF647 for B cells.

34 Use a razor blade **(SHARPS RISK)** to carefully remove the identified area from the resin with approximately 250 μm of excess resin on each side.
35 Mount the excised block on a metal pin using silver epoxy which is polymerised at 60°C for 1 h (CW2400, Circuit works)^2^.
36 Remove the sample from the oven and trim any excess resin and silver epoxy using a glass knife on an ultramicrotome (EM UC7).

<**CRITICAL STEP>** The resin block needs to be shaped into a square with one corner removed so that it is asymmetric, to aid in orientation of the block in the serial block face scanning electron microscope (SBF-SEM).

37 Finally, trim the block face at ultramicrotome position 0,0,0, until the tissue is reached.
  **<CRITICAL STEP>** Cutting empty resin from surface of block is essential to reduce time on the microscope. It also reduces charge build up at the start of the microscope run.
38 Sputter coat the block with 10 nm of platinum (Q150S, Quorum Technologies, Lewes, UK)
  **<CRITICAL STEP>** It is important to sputter coat the block, because it helps dissipate charge build up on samples. It also makes loading of samples into SBF-SEM easier, as it gives the sample surface a reflective surface for knife alignment).
39 Load the block into the SBF-SEM, which consists of a 3View2XP attached to a Sigma VP SEM with focal charge compensation (FCC). The SBF-SEM is used as a ‘smart trimming’ tool, allowing for the visual assessment of the tissue structure in real time.
40 Acquire backscattered electron detector images (3View detector, Gatan, Pleasanton, CA) and match with the structures imaged in the 63X confocal Z-stack.
41 When the Z-plane in the SBF-SEM contains the specific ROI, remove the sample from the microscope, re-trim the block using a glass knife to a sub-area of the ROI approximately 400 μm by 200 μm and 200 nm sections from the block face using an ultramicrotome (EM UC7) and a 6 mm histological diamond knife.
  <CRITICAL STEP> The SBF-SEM block shape is not optimal for regular sectioning methods. To ensure a clean collection of the region of interest, it is important to retrim the block.
42 Carefully deposit the sections onto silicon wafers using an eyelash tool and dry them on a hotplate at 70°C for 10 min.
43 Mount the silicon wafers onto SEM stubs using adhesive carbon tabs and load into a Quanta FEG 250 SEM.
44 Acquire tiled images of the whole section using MAPS software (version 1.1.8.603) with a low voltage high-contrast backscattered electron detector. Images are acquired using an accelerating voltage of 2.5 kV, a spot size of 3, a dwell time of 5 ms, a working distance of 6 mm and a pixel resolution of 10 nm.
45 Export individual images from the tiled sequence to tiff and align into a single image using the TrackEM2 plugin in Fiji 64^29^.

<**CRITICAL STEP>** A video guide prepared by our team for the workflow up to the SBF-SEM smart trimming is available for further reference here – https://vimeo.com/687907249.

### NanoSIMS acquisition *Timing 1 week*

**<CRITICAL>** Both the exported single image and the corresponding resin section on a silicon wafer can now be sent to guide NanoSIMS imaging and analysis. Since NanoSIMS analysis is destructive, it is essential that sections were thick enough to survive the entire analysis, yet still provide adequate counting statistics. Comprehensive details on sample preparation and data acquisition can be found in the literature^28^ and is summarised here. The samples are stable at room temperature at this stage and can be stored indefinitely before analysis.

**Figure 4:**
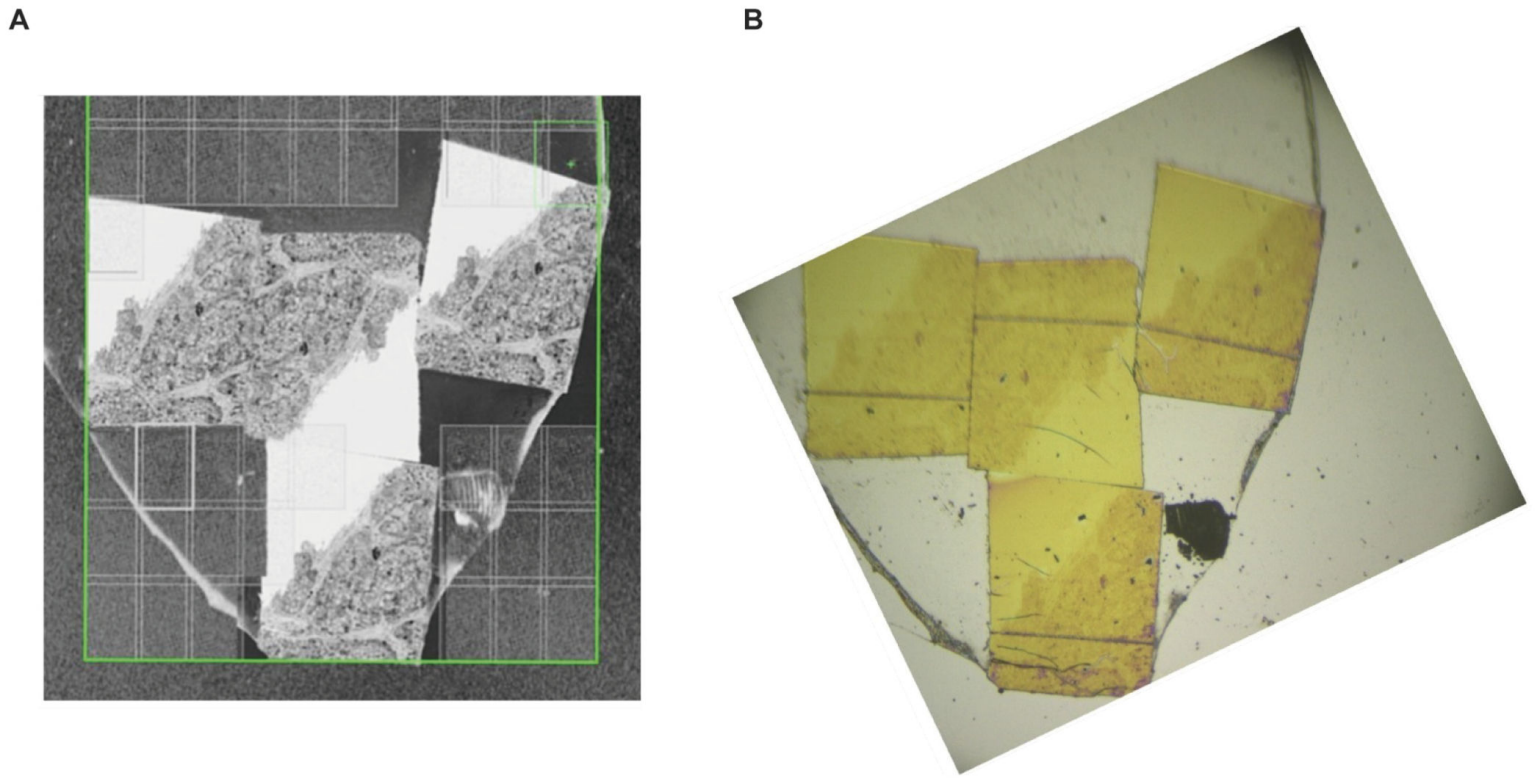
Correlating SEM images for NanoSIMS acquisition. (A) SEM image acquired at high pixel resolution enabling easy digital zooming for fine detail of tissue structure. (B) Corresponding optical image obtained using NanoSIMS CCD camera showing microtomed thin sections and structure within.

46 Position the fixed detector 7 on the NanoSIMS 50L to measure ^32^S. This will fix the value of the magnetic field in the mass analyzer.
47 Move detectors 1-6 to positions where they will measure ^12^C, ^16^O, ^12^C^14^N, ^12^C^15^N and ^31^P.
48 Operate the instrument in “Combined Analysis” mode to enable the additional detection of ^13^C^14^N.
49 Acquire the mass images at a 25 μm x 25 μm field width with a 150 μm D1 aperture (D1-4) to provide a mosaic correlating with regions previously analysed by fluorescence and scanning electron microscopy.
50 Load the sample into the UHV (1.5 x 10^-10^ torr) analysis chamber and move it to a location where it may be imaged optically using a CCD camera. The images provided by this camera can show structure from the sections that can easily be correlated with the corresponding SEM images of the same sections (Figure 4A - optical image from NanoSIMS and Figure 4B - SEM images).
51 The position of the ion beam for eventual NanoSIMS imaging is displayed as a cross on the optical image. Move the stage from its current position to that where the NanoSIMS imaging will take place.
52 Examine higher resolution SEM images with the cells of interest (B cells and CD8 T-cells) identified by confocal fluorescence microscopy. Tumour cells demarcated based on their cellular morphology are denoted^30^, as in Figure 2A.
53 Measure the Pulse Height Distributions (PHDs) of the electron multiplier (EM) detectors to ensure that their response is equivalent.
  - These should be measured on a section of tissue away from the actual area of interest.
  - Ensure that the PHDs are measured on samples that have been fully implanted, that is, the count rate measured on the detector has plateaued at a maximum value and remains constant.
  - After this calibration is complete, the count rate should be the same on adjacent detectors used to measure specific isotope ratios.

**<CRITICAL STEP>** Measuring PHDs is essential to allow measurement of accurate isotope ratios.

54 Align the secondary ion beam to ensure maximum signal reaches the detectors.
55 Use the quadrupole lens (Q) and the LF4 slit lens to optimize the mass resolving power in the high mass resolution spectra (HMRs) to ensure the correct mass peaks are measured without contributions from potential neighbouring mass interferences (eg. ^13^C and ^12^C^1^H).
56 Select image acquisition parameters: pixel resolution, dwell time, and number of planes.
  - The pixel size is selected to obey the rule of thumb that the spot size of the primary ion beam should be approximately twice the pixel size to prevent excessive over-and under sampling.
  - Multiple plane image scans provide a means for drift correction and sufficient counting statistics to improve precision in the ratio value. The minimum number of planes selected for the example experiments was 50 and the maximum, 150.

**<CRITICAL STEP>** It is essential to optimize the mass resolution, as contributions from mass interferences to the heavy isotope label masses can produce erroneously and misleadingly high isotope ratio values.

### NanoSIMS analysis *Timing variable*

**Figure 5:**
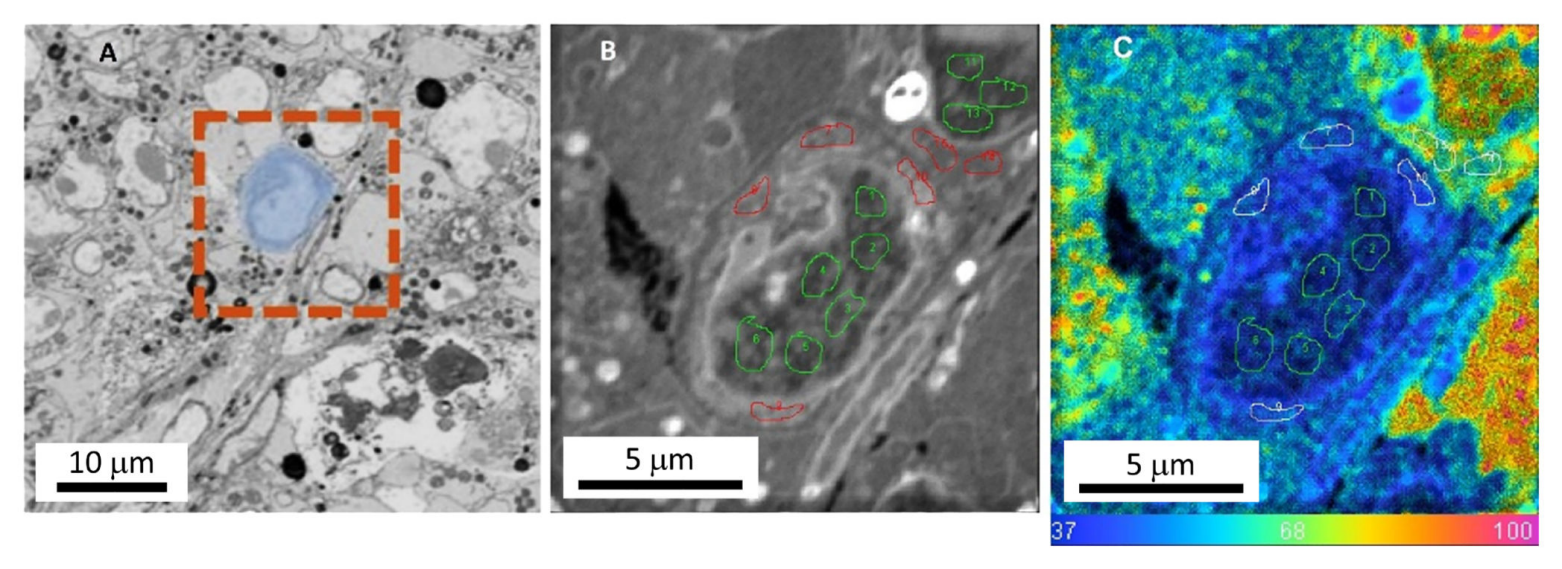
Example of ROI selection for quantitative analysis. (A) SEM image showing a B-cell (blue ROI). (B) ^12^C^14^N NanoSIMS image showing ROIs defined in nucleus of the B-cell (green ROIs) and cytoplasm (red ROIs). Similar ROIs are shown for a cancer cell in the top right of the image. (C) Corresponding nitrogen isotope ratio image. ^12^C^15^N/^12^C^14^N Isotope ratio images displayed as an HIS transformation. The blue hue represents the natural abundance ratio which is 0.37%.

57 Extract the processed images and quantitative data using the OpenMIMS plug-in for Fiji/ImageJ^31^.
  **<CRITICAL >** NanoSIMS raw data consists of mass resolved isotopic images from the eight defined masses. Carbon and nitrogen isotope images are derived from the ^13^C^14^N, ^12^C^14^N, and ^12^C^15^N images as ^13^C^14^N/^12^C^14^N and ^12^C^15^N/^12^C^14^N, respectively.
58 Draw ROIs within the nucleus and cytoplasm of the different cell types (B cells and CD8 T-cells) using the SEM image and ^12^C^14^N image as a guide. Figure 5 illustrates an example of ROI selection from a B-cell identified in the SEM image (Figure 5A). This image shows excellent tissue structure and allows us to define ROIs without any bias that may be elicited by the isotope ratio image (Figure 5B and C).
59 From each ROI, extract the total counts for all mass images, as well as the isotope ratios. The isotope ratio values can be extracted in two ways (using a nitrogen isotope ratio as an example):

- you can calculate the isotope ratio at each pixel within the measured ROI and the mean value, or
- the sum of the ^12^C^15^N and ^12^C^14^N counts over all pixels within the ROI and derive a simple ratio of those values.

These values should be similar unless the ratio values are disparately and heterogeneously distributed throughout the ROI, or the count rate of the heavy isotope is low.

## Timing

### Hydrodynamics-based transfection of DNA in the liver to generate liver tumours

Steps 1-2, hydrodynamics-based tail vein injection ˜2 h

Step 3, monitoring for liver tumours, 3-4 weeks

### Antibody clean-up

Steps 4-7, ˜20 min

### Stable isotope labelling and in vivo administration of antibodies

Steps 8-10, administration and preparing for infusion ˜1 h

Steps 11-14, infusion and tissue collection ˜4 h

### Embedding for EM and immunofluorescence imaging

Steps 15, overnight fixation ˜12 h

Steps 16 & 17, vibratome sectioning ˜2 h

Steps 18-21, confocal imaging ˜4h

Steps 22-32, embedding ˜3 d

### Targeted single section large area montaging

Steps 33-45, SBF-SEM smart trimming, 2-3 d depending on area of interest

### NanoSIMS acquisition

Steps 46-56, can roughly take maximum of 1 week, depending on the size and number of the regions

### NanoSIMS analysis

Steps 57-59, variable depending on the expertise of the user.

## Troubleshooting

Troubleshooting advice can be found in Table 2.

## Anticipated results

The results from the NanoSIMS analysis gives you the measure of the heavy isotope to natural isotope ratio. The ratios can be measured for each selected ROI within a selected region of a cell or the whole cell. In Keruzaler at al^1 12^C^15^N/^12^C^14^N isotope ratio derived from [amide-^15^N] glutamine and the ^13^C^14^N/^12^C^14^N isotope ratio derived from [U-^13^C] glucose were measured for each cell in either red or green clone of mixed-clone mammary gland tumours demonstrating strong [amide-^15^N] labelling in nucleoli, consistent with glutamine’s role in nucleotide biosynthesis, and higher [U-^13^C] labelling in the cytosol, reflecting glucose’s role as a carbon donor. Notably, green clones showed significantly greater incorporation of both glucose and glutamine labels than red clones, even in closely intermingled tumour regions^1^. In the liver tumour experiment ^12^C^15^N/^12^C^14^N isotope ratio derived from [amide-^15^N] glutamine and the ^13^C^14^N/^12^C^14^N isotope ratio derived from [U-^13^C] glucose were measured for ROIs within the nuclear and cytoplasmic components for the different cell types (B-cells and CD8 T-cells).

**Figure 6:**
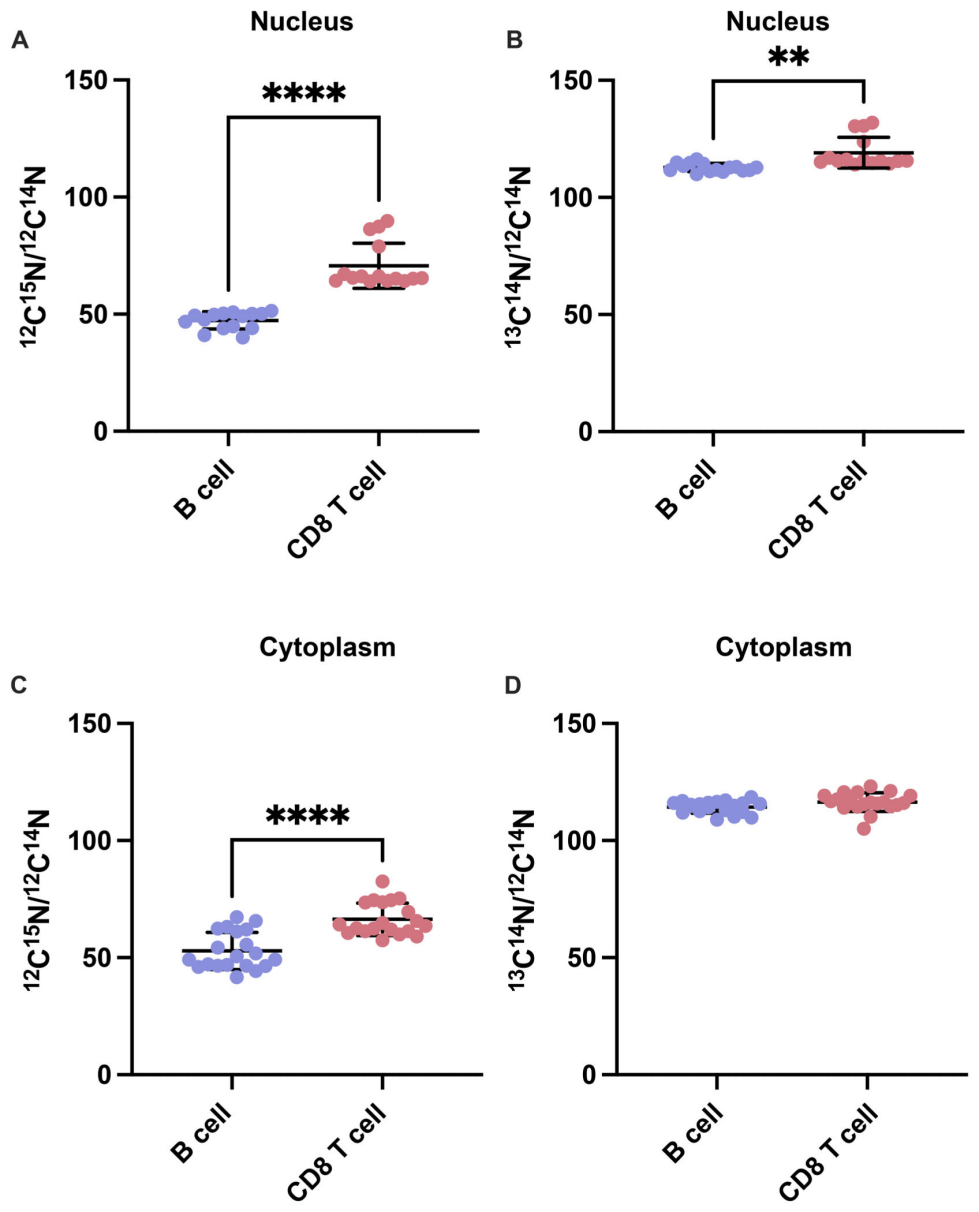
Enrichment of stable isotopes within the intracellular compartments of B cells and CD8 T cells. (A) Comparison of ^12^C^15^N/^12^C^14^N isotope ratio of ROIs from the nucleus of B cells and CD8 T cells (n=15 ROIs). (B) ^13^C^14^N/^12^C^14^N isotope ratio of ROIs from the nucleus of B cells and CD8 T cells (n=15 ROIs). (C) ^12^C^15^N/^12^C^14^N isotope ratio of ROIs from the cytoplasm and (D) ^13^C^14^N/^12^C^14^N isotope ratio of ROIs from the cytoplasm of B cells and CD8 T cells (n=20 ROIs). Statistical significance was assessed by unpaired t-test (mean +/- SD,**p<0.01, ****p<0.0001).

As described in the previous section (step 51) the sum of counts for each of the following isotopes, ^12^C^15^N, ^12^C^14^N, ^13^C^14^N and ^12^C^14^N, per pixel within each of the ROIs are calculated. A simple ratio of ^12^C^15^N/^12^C^14^N and ^13^C^14^N/^12^C^14^N is obtained for the nuclear and cytoplasmic ROIs of B-cells and CD8 T-cells (Figure 6). Similar results can also be obtained for other cellular compartments such as mitochondria, nuclear membrane, etc and other nutrients labelled with stable isotopes if the corresponding ROIs can be traced using SEM image and natural abundances isotope image as a guide to identify the correct components. For measuring the ratios in other cell types the respective fluorescent-tagged antibodies need to be administered. We have successfully tested antibodies for macrophages and neutrophils. Although the major limitation of these results is that it does not provide an absolute quantification of the amount of ^13^C or ^15^N and is only a comparison of the ratios of ^12^C^15^N/^12^C^14^N and ^13^C^14^N/^12^C^14^N, it allows us to precisely trace the fate of any stable isotope labelled nutrient at a subcellular level.

## Figures and Tables

**Table 1:** Advantages and limitations of recently developed metabolic profiling methods.

Method	Advantages	Limitations
SCENITH^11^ - Flow Cytometry-Based Method to Functionally Profile Energy Metabolism with Single-Cell Resolution	Single cell resolution. Can profile both abundant and non-abundant cell types in parallel. Can be used to directly profile metabolism *ex vivo* from patient samples.	Limited insight into metabolic functions of individual cells. Spatial information of tissue histology is lost.
scMEP^12^ - single-cell metabolic regulome profiling	Single cell resolution. Robust identification of different cell types. Imaging-based scMEP allows for study of spatial organisation of cellular metabolism.	Offers an approximation of metabolic states, cannot be used to study metabolic fluxes.
OrbiSIMS^13,14^ - Label-free metabolic imaging with subcellular lateral resolution and high mass-resolving power	Single cell resolution High mass resolving power and high spatial resolution. Minimal sample preparation.	Offers better coverage of apolar metabolites. Cell type identification with IHC cannot be carried out on the same sample.
DESI-MSI + IF^15^ - Cell-Type-Specific Metabolic Profiling by Combining Desorption Electrospray Ionization Mass Spectrometry Imaging and Immunofluorescence Staining	Offers a large coverage of both polar and apolar metabolites. Can be used to study spatial changes in metabolic fluxes within and across different tissues.	Relatively low resolution Not recommended for studying the metabolism of rare cell populations.

**Table 2:** Troubleshooting

Step	Problem	Possible reason	Possible solution
18	DAPI signal is not detected or is present on the edges of the tissue	The DAPI did not penetrate through the section	Consider longer incubation with DAPI on a gentle shaker
39	Still seeing charge in the sample despite setting up the FCC	FCC is not positioned correctly for the ROI	Re-vent the microscope and adjust the positioning of the FCC in relation to the ROI.
40	Signal on the backscatter detector is lower than expected and imaging is difficult	Contrast in the sample is not sufficient	Adjust staining steps on the embedding process to increase contrast in the sample.
53	Count rate not the same as measured on two adjacent detectors to be used to derive isotope ratios	C4X deflector not set properly.	Scan voltage across C4X while simultaneously measuring all mass signals. Set C4X voltage value where it is situated on the plateau for ALL masses. It does not necessarily need to be centered within the plateau.
55	Unable to measure natural isotope ratio values on control sample.	Poor mass resolution, detectors not properly calibrated, C4X deflector not set properly.	Check C4X deflector value first as above as it is a quick test and could be the solution. Recheck PHDs as per step 46. Consider using a narrower entrance slit and/or aperture slit.

## Data Availability

The raw images associated with the SEM and NanoSIMS for the different cell types (Figure 2) are available at https://doi.org/10.25418/crick.24989841.

## Editorial Summary

Evaluating the metabolic organisation of complex tissue microenvironments is useful for understanding cellular function within tissues. This protocol describes a multimodal imaging pipeline for cell type identification and stable isotope imaging.

## Key points

- To understand complex tissues, it is useful to map metabolic processes to specific cell types. Nanoscale secondary ion mass spectrometry (NanoSIMS) can differentiate between isotopes derived from labeled metabolites at sub-cellular resolution.
- This protocol combines three imaging modalities: confocal microscopy of fluorescent probes (antibodies); electron microscopy; and NanoSIMS enabling cell type identification, characterization of the cellular architecture, and extraction of metabolic information.

## References

[1] Kreuzaler P (2023). Vitamin B5 supports MYC oncogenic metabolism and tumor progression in breast cancer. Nat Metab.

[2] Maclachlan C, Sahlender DA, Hayashi S, Molnár Z, Knott G (2018). Block Face Scanning Electron Microscopy of Fluorescently Labeled Axons Without Using Near Infra-Red Branding. Front Neuroanat.

[3] Fearns A, Greenwood DJ, Rodgers A, Jiang H, Gutierrez MG (2020). Correlative light electron ion microscopy reveals in vivo localisation of bedaquiline in Mycobacterium tuberculosis–infected lungs. PLoS Biol.

[4] de Boer P, Hoogenboom JP, Giepmans BNG (2015). Correlated light and electron microscopy: ultrastructure lights up!. Nat Methods.

[5] Arlauckas SP (2017). In vivo imaging reveals a tumor-associated macrophage–mediated resistance pathway in anti–PD-1 therapy. Sci Transl Med.

[6] Greenwood DJ (2019). Subcellular antibiotic visualization reveals a dynamic drug reservoir in infected macrophages. Science (1979).

[7] Méndez-Lucas A (2020). Identifying strategies to target the metabolic flexibility of tumours. Nat Metab.

[8] Quinn A (2024). Host-derived organic acids enable gut colonization of the honey bee symbiont Snodgrassella alvi. Nature Microbiology.

[9] Lechene CP, Luyten Y, McMahon G, Distel DL (2007). Quantitative Imaging of Nitrogen Fixation by Individual Bacteria Within Animal Cells. Science (1979).

[10] Loussert-Fonta C (2020). Correlation of fluorescence microscopy, electron microscopy, and NanoSIMS stable isotope imaging on a single tissue section. Commun Biol.

[11] Argüello RJ (2020). SCENITH: A Flow Cytometry-Based Method to Functionally Profile Energy Metabolism with Single-Cell Resolution. Cell Metab.

[12] Hartmann FJ (2021). Single-cell metabolic profiling of human cytotoxic T cells. Nat Biotechnol.

[13] Passarelli MK (2017). The 3D OrbiSIMS—label-free metabolic imaging with subcellular lateral resolution and high mass-resolving power. Nat Methods.

[14] Kern C (2024). Orbi-SIMS Mediated Metabolomics Analysis of Pathogenic Tissue up to Cellular Resolution. Chemistry–Methods.

[15] Yan X (2020). Cell-Type-Specific Metabolic Profiling Achieved by Combining Desorption Electrospray Ionization Mass Spectrometry Imaging and Immunofluorescence Staining. Anal Chem.

[16] Kreuzaler P (2019). Heterogeneity of Myc expression in breast cancer exposes pharmacological vulnerabilities revealed through executable mechanistic modeling. Proc Natl Acad Sci U S A.

[17] Siuzdak G (2023). Subcellular quantitative imaging of metabolites at the organelle level. Nature Metabolism.

[18] Pfister D (2021). NASH limits anti-tumour surveillance in immunotherapy-treated HCC. Nature.

[19] Kotsiliti E (2023). Intestinal B cells license metabolic T-cell activation in NASH microbiota/antigen-independently and contribute to fibrosis by IgA-FcR signalling. J Hepatol.

[20] Garnelo M (2017). Interaction between tumour-infiltrating B cells and T cells controls the progression of hepatocellular carcinoma. Gut.

[21] Yuneva MO (2012). The Metabolic Profile of Tumors Depends on Both the Responsible Genetic Lesion and Tissue Type. Cell Metab.

[22] Tao J (2015). Distinct anti-oncogenic effect of various microRNAs in different mouse models of liver cancer. Oncotarget.

[23] Lechene C (2006). High-resolution quantitative imaging of mammalian and bacterial cells using stable isotope mass spectrometry. J Biol.

[24] Titze B, Genoud C, Friedrich RW (2018). SBEMimage: Versatile Acquisition Control Software for Serial Block-Face Electron Microscopy. Front Neural Circuits.

[25] Bogovic JA, Hanslovsky P, Wong A, Saalfeld S (2016). Robust registration of calcium images by learned contrast synthesis.

[26] (2010). Valap for agar mounts. Cold Spring Harb Protoc.

[27] Deerinck T (2010). NCMIR methods for 3D EM: a new protocol for preparation of biological specimens for serial block face scanning electron microscopy.

[28] McMahon G, Lechene CP (2021). High-Resolution Multi-Isotope Imaging Mass Spectrometry (MIMS) Imaging Applications in Stem Cell Biology. Curr Protoc.

[29] Cardona A (2012). TrakEM2 Software for Neural Circuit Reconstruction. PLoS One.

[30] de Senneville BD (2021). Deciphering tumour tissue organization by 3D electron microscopy and machine learning. Commun Biol.

[31] Schindelin J (2012). Fiji: an open-source platform for biological-image analysis. Nat Methods.

